# Sudden Death from Plugging of the Pulmonary Veins in a Pregnant Lady

**Published:** 1854-03

**Authors:** 


*Sudden Death from Plugging of the Pulmonary Veins in a Pregnant Lady.* By E. Smith, M.D. L.L.B.

This case is one of great rarity and interest, and its value is enhanced by the accompanying comments.

"On Sunday evening, April 24th, I was urgently summoned to see a lady who was reported to be delirious. On arriving at the house, I found that she was dead, and had been so fully twenty minutes. She was a patient of Mr. Bartlett and Dr. Jackson, of Notting Hill, and was altogether unknown to me. I found that she was about twenty years of age, a little above the middle size, well developed, and in good condition, and within a few days of the term of utero-gestation of a second child. She had been perfectly well until within ten minutes of her death, except that she had complained of some pain and tenderness on the inner side of the left thigh, and to relieve this, had been directed to lie in a recumbent position. She had eaten a very hearty dinner at three P. M., and tea at six P. M., and was full of spirits throughout the day, and up to nearly eight P. M. She had worn the stays used by pregnant ladies, even when lying upon the bed, contrary to the directions of her medical adviser; and it is probable that they were well laced. The child was known to be alive on Saturday evening, but nothing could be learned as to its vitality on Sunday. While lying upon the bed, dressed, and with her stays on, and in excellent spirits, she suddenly uttered a shriek, and flung her arms about wildly, and cried, "Oh, my head! I cannot breathe! I am going mad!" and also, "Give me my breath!" This continued for about five minutes, during which time her hand was placed upon her chest; and then she became calm for a moment, and said to her husband, "There, Charles, I am better," and expired. The face was deeply livid, and the body bent, so that the chin approached her knees. When I saw her, the face was blanched, and she lay stretched on the bed. Having learned several of these particulars within a few minutes after my arrival, I became anxious as to the propriety of performing the Cæsarian section, to save the child; but, since so long a period had already elapsed after the death of the mother, since I had neither stethoscope nor scalpel with me, having been summoned from church, since, moreover, I knew nothing of the case previously, and could not fully persuade the husband and friends of the reality of their loss, I determined not to perform it.

By the kindess of Mr. Bartlett, I had the opportunity of assisting Dr. Jackson and himself at the *post-mortem* examination, forty hours after death, and of making the requisite microscopic investigation of the tissues. The features had lost somewhat of their pallor, and a fluid, very slightly sanious, was exuding from the mouth and nostrils. The under part of the body, as it lay on the table, was not only greatly congested, but presented many well-marked, purplish-black petechiæ. The left leg was not swollen or inflamed. The blood was black, and fluid universally, except in the pulmonary veins, where the whole tube was filled by a cylinder of coagulum, having a central clot of blood, inclosed by two layers of condensed fibrin, the outer one of which was colorless, and the whole so firm in texture, that it could be handled and pressed with impunity. It was not strongly adherent to the lining membrane of the vein. The number of white corpuscles was considerably beyond the normal standard. The heart was flaccid, and rather enlarged on the right side. The tissue was undergoing the process of granular degeneration, or the first step of the process of fatty degeneration, and more particularly on the right side. The left side was empty—without coagula, even. The right ventricle contained, and the right auricle was distended with, fluid black blood. The valves were healthy. The arteries were preternaturally small, so much so that the aorta at its bifurcation could not admit the end of a small little finger, and the capacity of the external iliac was not greater than that of a swan's quill. Neither blood nor coagula were found within any of them, nor were any of them ruptured. The veins were immensly and universally distended, and appeared to be as much larger as the arteries werc smaller than the natural size. The inferior cava was fully an inch and a quarter in diameter. The most remarkable enlargement, however, was in the ovarian veins; but whether this enlargement was greater than is usual at the full term of utero-gestation, before labor has commenced I cannot tell. They were about twelve inches in length by three-quarters of an inch in breadth, and passed in a curved direction from the ovarian plexus in the broad ligaments, along the iliac fossæ, to the front of the vena cava on the right, and to the renal vein on the left side. The left was the larger of the two. The right one had thinner coats, so that the dark blood withn it was more evident, and terminated by an opening so constricted that a crow-quill could scarcely be introduced into the vessel from the vena cava. There was a bulging of the vessel directly on the side of the vena cava, viz., close to the constricted opening into the cava; and the trunk of both vessels was of even diameter throughout. A careful examination showed that the inner coat of these veins had not given way. The stomach and intestines were enormously distended with flatus, and contained fecal and partly-digested matter. There was no odor of hydrocyanic acid. The uterus was normally developed and entire, but its parietes were flaccid. The placenta was very readily detached, and was bloodless, and had not undergone the degenerative process. The membranes were unbroken, and the os uteri perfectly closed. The child (a male) was somewhat small, and the cuticle peeled from the subjacent parts on very slight pressure; but there were no other signs of commencing decomposition. The ovaries were healthy. The diaphragm was pushed upwards to the level of the fourth or fifth ribs, thus greatly diminishing the capacity of the thorax. The lungs were much collapsed, and crepitus on pressure was but slight. Numerous bubbles of extravasated air were scattered over the surface, directly under the visceral layer of the pleura, and more particularly on the left lung, towards the base. The discoloration on the posterior and inferior aspects was much greater than is usually met with as a *post-mortem* occurrence. The tissue was somewhat readily broken upon pressure, but no rupture of the structure was evident. It contained very many granular corpuscles; but, since the blood was fluid, with no appearance of pus, and contained, in other parts, an unusual quantity of white corpuscles, it is probable that these cells were not exudation cells, but white corpuscles of the blood. The pleural cavity, on the left side, contained about three ounces of a deeply tinged sanious fluid, without coagula. On the right side, the quantity was smaller, and the fluid less discolored. The sinuses and larger veins of the brain were very turgid. The substance of the brain was of normal consistence, and had not been lacerated; it was slightly congested. There was no effusion at the base, or in the ventricles of the brain, neither any remarkable congestion of the choroid plexus. The tissues throughout the body indicated a somewhat unusual degree of flaccidity.

On a review of the symptoms and *post-mortem* signs, the following thoughts naturally occur to the mind: The mode by which death supervened was that of suffocation. The general flaccidity of the tissues, with the degenerative process proceeding in the center of the circulating sysem, and the presence of an increased quantity of white corpuscles in the blood, indicate an atonic condition of system, one especially liable to take on deranged nervous action, and likely to succumb under the influence of a violent shock. May the enlargement of the veins be in any degree attributed to the diminished size of the arteries? The venous congestion was probably of some duration, and accompanied or caused by the absence of the accustomed degree of bodily exercise, the horizontal position in which she had of late indulged, the large size of the veins, the condition of the blood, the pressure of the gravid uterus, and the lacing of the stays. The congestion would be greatly increased, probably, by the two hearty meals which had been taken within the four and a half hours preceding the death, and the enormous distension of the intestinal canal. The extravasation of air under the pleura, the injection of the parenchyma of the lung with fluid venous blood, and the petechiæ on the skin, would be due to the violent death-struggles. The effusion of bloody fluid into the pleural cavity would arise from the last-mentioned cause, added to those of the fluidity of the blood and the congestion of the lungs. The cause of the fluidity of the blood is not very evident; but it may be owing to a combination of three attendant circumstances, viz., the condition of the blood, the rapidity of the process of dying, and the suffocation. The special exception to the fluidity of the blood observed in the plugging up of the pulmonary veins by coagula, accompanied by great distension of the venous system, and the venous side of the heart, and the emptiness of the arterial system and left side of the heart, cannot but attract attention. I am fully impressed with the inherent difficulty attending the solution of the problem, as to how far the formation of such coagula may be simply an attendant occurrence of the act of dying, or how far the coagula should be regarded as giving rise to those symptoms which indicate approaching death; that is to say, whether they be really a cause or an attendant of the act of dying. Since coagula are so frequently found as dying or *post-mortem* occurrences, we cannot but regard with suspicion any opinion favoring the supposition, that, under any circumstances, they are true causes of death. Without being dogmatical, however, I am inclined to think that the special exception formed by them in this case, the fact that the clot had time to form two envelopes of condensed fibrin, the outer one of which was quite free from the presence of red corpuscles, in a death so sudden and rapid, would almost suffice to induce us to regard them as a cause, and not an attendant occurrence of the death. The greatly diminished capacity of the chest, induced somewhat suddenly, perhaps, by the distension of the stomach and intestines, would impede the action of the lungs, and, by lessening the quantity of inspired air, cause a retardation of the sanguineous current, and thus tend to the formation of the coagula. If this view be a correct one, we may readily account for the sudden origin of violent and fatal symptoms. Without such an explanation, while I can see abundant cause for death, I cannot find the occurrence which gave rise to the fatal symptoms at a distance of two hours from the last meal, and not an hour and a half earlier.—*Medical Times and Gazette.*

